# Modulation of the Sporulation Dynamics in the Plant-Probiotic *Bacillus velezensis* 83 via Carbon and Quorum-Sensing Metabolites

**DOI:** 10.1007/s12602-025-10482-w

**Published:** 2025-02-26

**Authors:** Esmeralda Yazmín Soriano-Peña, Agustín Luna-Bulbarela, Sergio Andrés Cristiano-Fajardo, Enrique Galindo, Leobardo Serrano-Carreón

**Affiliations:** Departamento de Ingeniería Celular y Biocatálisis, Instituto de Biotecnología, Universidad Nacional Autónoma de México, Av. Universidad 2001, C.P.62210 Cuernavaca, Morelos México

**Keywords:** *Bacillus velezensis*, Spore production, Overflow metabolites, CSF peptide, Quorum-sensing metabolites

## Abstract

Spore-forming Bacilli, such as the plant-associated *Bacillus velezensis* strains, are widely used as probiotics, known for their safety and substantial health benefits for both animal and plant species. Through differentiation pathways mediated by quorum-sensing metabolites (QSMs), these bacteria develop multiple isogenic subpopulations with distinct phenotypes and ecological functions, including motile cells, matrix-producing*/*cannibalistic cells, competent cells, spores, and others. However, the heterogeneity in *Bacillus* populations is a significant limitation for the development of spore-based probiotics, as nutrients supplied during fermentation are consumed through non-target pathways. One of these pathways is the generation of overflow metabolites (OMs), including acetoin and 2,3-butanediol. This study elucidates, using a 2^3^ full factorial experimental design, the individual effects of OMs, QSMs, and their interactions on the sporulation dynamics and subpopulation distribution of *B. velezensis* 83. The results showed that OMs play a relevant role as external reserves of carbon and energy during in vitro nutrient limitation scenarios, significantly affecting sporulation dynamics. OMs improve sporulation efficiency and reduce cell autolysis, but they also decrease cellular synchronization and extend the period of spore formation. Although QSMs significantly increase sporulation synchronization, the desynchronization caused by OMs cannot be mitigated even with the addition of autoinducer QSM pro-sporulation molecules, including competence and sporulation stimulating factor “CSF” and cyclic lipopeptides. Indeed, the interaction effect between OMs and QSMs displays antagonism on sporulation efficiency. Modulating the levels of OMs and QSMs is a potential strategy for regulating the distribution of subpopulations within *Bacillus* cultures*.*

**Supplementary Information:**

The online version contains supplementary material available at 10.1007/s12602-025-10482-w.

## Introduction

Spore-forming Bacilli, such as the plant-associated *Bacillus velezensis* strains, are widely used as probiotics, known for their safety and substantial health benefits for both animal and plant species [14, 69]. In agro-industry applications, these bacteria are highly valued for their ability to deliver biological control against fungal phytopathogens. Through the intricate control of genetic circuits and differentiation pathways mediated by quorum-sensing metabolites (QSMs), such as the pheromone ComX, Phr mature peptides, and lipopeptides (LPs), these bacteria develop multiple isogenic subpopulations with distinct phenotypes and ecological functions, including motile cells, matrix-producing*/*cannibalistic cells, competent cells, and spores [1, 42, 48–50, 65, 86, 91]. The heterogeneity of *Bacillus* spp. populations enables its successful establishment and persistence in natural settings, for instance, by the biofilm formed during interactions with plant roots. However, from the bioprocess point of view, this represents a significant limitation for the development of spore-based probiotics, as nutrients supplied during fermentation are consumed through non-target pathways. One of these pathways is the generation of carbon overflow metabolites (OMs) during vegetative growth, including acetoin and 2,3-butanediol (2,3-BD). Interestingly, in *Bacillus velezensis* and related strains, OMs act as external carbon reserves, which are consumed during sporulation [3, 12, 13, 52, 98]. This research explores how the subpopulation distribution (vegetative cells, lysed cells, and spores) and sporulation profiles in *Bacillus velezensis* 83, a wild-type strain with biocontrol applications, are influenced by OMs and QSMs produced during cell growth.

*B. velezensis* 83 is a bacterium isolated from the mango phyllosphere used as a plant-probiotic, with antibiosis capacity against phytopathogenic fungi and the ability to promote growth and plant root system development [6, 55]. Integrative omics revealed its potential to synthesize antimicrobial metabolites including non-ribosomal peptides and polyketides, as well as phytostimulants including biopolymers and acetoin [5, 54]. Characterized by its ability to produce significant levels of thermoresistant spores, this strain enables the development of solid formulations useful for crop protection with a prolonged shelf life, making it an interesting platform for studies on cellular differentiation and technological development [12, 23]. *B. velezensis* 83 and the model strain *B. subtilis* 168 share a high similarity in genes associated with the signaling and morphogenesis of sporulation [5]. Consequently, the comprehensive understanding of *B. subtilis* 168 serves as an essential foundation for research on other similar species. Nonetheless, there are important differences that need to be emphasized. *B. velezensis* 83 shows a unique configuration of Rap-Phr cassettes, operons regulating a phosphorylation cascade involved in sporulation, distinguished by the presence of at least 4 Rap (response-regulator aspartyl phosphate) phosphatases and 3 corresponding Phr (phosphatase regulator) peptides, a pattern that varies among species [24]. Unlike the domesticated *B. subtilis* 168 strain, *B. velezensis* 83 is a plant-associated strain with an active DegS-DegU system, enabling the production of poly-γ-glutamic acid (γ-PGA) and non-ribosomal cyclic lipopeptides such as bacillomycin D [5, 12, 41, 89]. This distinction is crucial when using *B. velezensis* 83 as a model for the characterization and production of spore-based probiotics. The synthesis of γ-PGA diverts carbon from biomass production and increases the viscosity of culture media, leading to mixing challenges and oxygen limitations, which adversely affect growth, sporulation, and subsequent recovery operations [76].

Molecular research in other model strains has highlighted the significance of both QSMs and OMs in sporulation, offering useful knowledge for the design of new probiotics production processes. Studies usually employ non-producing mutants and exogenous induction to understand the effects of specific molecules on sporulation. The activation of infrequent cellular states, such as competence, is possible in *Bacillus* mutant strains [91]. During *Bacillus* spp. growth, a range of secondary metabolites, including QSMs (*e.g.*, ComX pheromone and Phr mature peptides), non-ribosomal peptide antibiotics (surfactin, bacillomycin D and others), and polyketides, are released into the culture medium, directly impacting the process of sporulation [84]. For instance, surfactin mediates signaling cascades in quorum-sensing (QS) responses, promoting the differentiation of *Bacillus* spp. populations into multiple types including non-motile cells, matrix-producing*/*cannibalistic cells, and spores [48, 49, 56, 78]. This lipopeptide promotes differentiation pathways in *B. subtilis* by inducing potassium leakage and activating the membrane protein kinase KinC [47]. *Bacillus subtilis* mutants affected in *srfAA*, which do not produce surfactin, show a significant reduction in spore frequency, highlighting the importance of this lipopeptide [67, 84]. The addition of spent culture supernatant of *Bacillus* broths can restore sporulation [84]. In line with this, *Bacillus amyloliquefaciens* mutants with a defective *srfA* operon show deficiencies in biofilm formation and sporulation, which can be recovered through external stimulation with surfactin [10]. In a similar manner, bacillomycin D, a lipopeptide produced by *Bacillus amyloliquefaciens* (currently recognized as *Bacillus velezensis*), performs similar roles. Bacillomycin D and Mn^2+^ promote spore formation through stimulation of *kinB* and *kinD* transcription [100] and modulate biofilm formation through the KinB-Spo0A-SinI-SinR pathway by promoting iron acquisition [96, 97].

Regarding the OMs production in *Bacillus*, acetoin and 2,3-butanediol (2,3-BD) are glucose metabolism by-products with relevant roles in the biofilm formation and sporulation processes. These OMs act as an oxidation–reduction buffer for NAD/NADH interconversion and provide carbon skeletons for amino acid synthesis [3, 73, 88]. Acetoin production is essential for biofilm formation and functions as an extracellular pH buffer [75]. The genes necessary for their biosynthesis and catabolism are known [46, 73], but not all the involved transporters are identified. Inactivation of the putative acetoin transporter *ytrABCDEF* in *B. subtilis* leads to reduced consumption of this metabolite during the stationary phase, lower viable cell concentration, and decreased sporulation frequency (defined as [Heat-resistant CFU]/ [Viable cells]) [98]. Mutant strains of *B. subtilis* with impaired catabolite repression and capable of synthesizing acetoin dehydrogenase, even in the presence of glucose, sporulate at a higher frequency in glucose-supplemented medium compared to the wild-type strain [52].

Previous studies in *B. velezensis* 83 also indicated that the production of quorum-sensing (QSMs) and overflow metabolites (OMs) during the growth phase, as well as the subsequent utilization of OMs as an external carbon source, would have a significant impact on sporulation dynamics. *B. velezensis* 83 cultivations under acidic conditions (pH 5.0) results in complete inhibition of the sporulation process during the period of nutritional limitation, in contrast to near-neutral conditions (pH 6.8) [12]. The expression of genes encoding the pheromone ComX (during the growth phase) and the phosphatase regulator PhrC (during the stationary phase) consistently decrease significantly at pH 5.0, suggesting that the QSMs threshold is not achieved under these conditions. In fact, exogenous stimulation of *B. velezensis* 83 vegetative cells grown at pH 5.0 with synthetic competence and sporulation stimulating factor “CSF” or supernatant from cultures at pH 6.8 and high cell concentration (× 10^10^ cell/mL) restores sporulation, highlighting the role of QSMs in sporulation dynamics (*unpublished data*). Nevertheless, spores formed at pH 5.0 with exogenous CSF induction fail to mature, indicating that interaction with additional metabolites is required to complete the process. Among the potential factors are OMs or the synergistic action of other Phr peptides. About the former, during the growth phase of cultures at pH 5.0 (a condition that prevents sporulation), acetoin and 2,3-butanediol are accumulated to levels comparable to those at pH 6.8, where sporulation occurs normally. However, metabolites are poorly consumed at pH 5 even after glucose depletion. Interestingly, in cultures developed at pH 5.0, sporulation and specific consumption of OMs can be restored to normal levels during the glucose limitation period, by increasing the pH to 6.8 [12].

The development of improved bioprocesses for spore production of a biocontrol agent such as *B. velezensis* 83 is imperative to reduce production costs and enhance their competitive position relative to chemical pesticides. Strategies should prioritize the optimization of fundamental goal functions, specifically (a) cell yield, (b) sporulation efficiency, and (c) spore quality (heat resistance). Previous work by our research group have demonstrated that a combined strategy of pH adjustment (pH 5 and 6.8 during growth phase and sporulation, respectively), along with fed-batch processes, allows for the modulation of sporulation initiation and a significant increase in the final spore cell concentration by improving the cell yield, while avoiding γ-PGA production and the associated increase in viscosity. This strategy also facilitates downstream operations and positively impacts the feasibility of processes utilizing biopolymer-producing *Bacillus* strains [12]. Nevertheless, the cultures show low relative sporulation efficiency, primarily due to autolysis and prolonged differentiation phases. Strategies using molecular approaches to inhibit autolysis via the generation of null mutants of the involved genes were reported [80]. However, due to restrictions on the release of genetically modified microorganisms in plant-probiotic products, the use of mutant strains is not an option for commercial spore production,therefore, alternative strategies must be assessed. Exogenous stimulation, along with cultivation methods such as fed-batch processes, is proposed here. As previously mentioned, during the vegetative growth phase and prior to the onset of sporulation, OMs and QSMs, such as pheromone ComX, lipopeptides “LPs” (surfactin and bacillomycin D), and competence and sporulation stimulating factor “CSF,” are synthesized. Simultaneously with the increase in cell concentration and the accumulation of these metabolites, many cellular responses are activated. For instance, the accumulation of the ComX pheromone, a component of the ComQXPA signaling system, induces the transcription of genes responsible for surfactin synthesis in *B. subtilis* [17]. Nevertheless, there are no studies showing how interactions between these metabolites affect the dynamics of sporulation in *Bacillus velezensis*, particularly in terms of efficiency and half-sporulation time. This work presents sporulation efficiency as a stoichiometric parameter that quantifies the percentage of spore concentration in relation to the maximum cell concentration reached in a culture. Unlike sporulation frequency, which denotes the ratio of spores within a cell suspension (comprising only vegetative cells and spores), the sporulation efficiency considers the cell loss caused by autolysis as part of the metric. The proposed parameter emerged as a more proper variable for evaluating the balance of subpopulations in *B. velezensis*, considering that up to 60% of the cells generated in a culture might be lost as a result of lysis. Batch cultures were performed with different initial concentrations of cells and glucose in order to regulate the concentration of OMs and QSMs achieved during nutritional limitation. Subsequently, the individual effects of OMs, QSMs, and their interactions on the sporulation dynamics of *B. velezensis* 83 were elucidated through in vitro nutritional limitation scenarios, using a 2^3^ full factorial experimental design. Potential designs for rational fermentation strategies to enhance spore production are discussed.

## Materials and Methods

### Strain and Inoculum Preparation

*Bacillus velezensis* 83 was provided by Agro & Biotecnia S de RL de CV (Cuernavaca, Mexico) and is deposited at the Belgian Coordinated Collection of Microorganisms (BCCM, strain LMGS-30921). Spores preserved in 40% glycerol at − 20 °C were activated on Petri dishes containing YPG medium (g/L: 10 peptone, 10 yeast extract and 10 glucose) supplemented with agar (15 g/L). Subsequently, two subcultures were conducted in liquid YPG medium to generate a spore-free vegetative cell suspension, using 2 L Fernbach flasks, at 30 °C and 200 rpm for 12 h each. To remove quorum-sensing metabolites (QSMs) and other secondary metabolites (lipopeptides and carbon overflow metabolites) present in the suspension, cells in exponential growth phase were harvested by centrifugation (10 min, 3600 g) and subsequently resuspended in saline solution (0.9% NaCl and 0.05% Tween 80).

### Experimental Designs and Culture Conditions

#### 2^2^ Factorial Design

An experimental design 2^2^ was carried out to investigate the effects of initial glucose and cell concentrations on the production of carbon overflow metabolites, lipopeptides, and the sporulation dynamics in *B. velezensis* 83. The study employed low and high levels for the two independent variables: glucose concentration (Glc_0_ +  = 15 g/L, Glc_0_- = 0.15 g/L) and cell concentration (X_0_ +  = 1 × 10^9^ cells/mL, X_0_- = 1 × 10^7^ cells/mL), resulting in four distinct experimental scenarios (Fig. 2). The corresponding cultures were performed in a 3.0-L stirred-tank bioreactor (Dusher®, Mexico City, MX), using 1.8 L of mineral medium containing (g/L): 4 (NH_4_)_2_SO_4_, 5.32 K_2_HPO_4_, 6.4 KH_2_PO_4_, 0.4 MgSO_4_⋅7 H_2_O, 0.1 g CaCl_2_, 0.08 FeCl_3_·6H_2_O, 0.019 MnCl_2_·4H_2_O, and glucose as carbon source [13]. The reactor was equipped with three 6-blade Rushton turbines (impeller-to-tank diameter ratio “D/T” was 0.5, where the tank diameter “T” was 0.15 m). Operation was conducted at 30 °C with continuous aeration at 1 vvm. Dissolved oxygen tension (DOT) was kept ≥ 15% via PID (proportional–integral–derivative) control, regulating agitation speed from 200 to 700 rpm. Automatic titration with 2N NaOH solution was used to maintain pH at 6.8.

#### 2^3^ Factorial Design

A 2^3^ factorial experimental design was implemented to evaluate the individual and combined effect of three variables: (1) overflow metabolites (OMs, including acetoin and 2,3-butanediol), (2) lipopeptides (LPs, such as surfactin and bacillomycin), and (3) competence and sporulation stimulating factor “CSF” (PhrC mature peptide, a quorum-sensing metabolite), on the sporulation dynamics of *B. velezensis* 83. Experiments involved the exogenous addition of these molecules to vegetative cells in glucose-free mineral medium. Vegetative cells in the exponential phase were employed in these experiments, obtained from a nutrient-rich medium (YPG medium), to minimize the probability of harvesting cells that were activated in phosphorylation cascades and differentiated, as mentioned by Veening et al. [95]. In this nutrient-rich medium, *B. velezensis* 83 failed to generate spores before 96 h of cultivation. Before inoculation, the obtained cells were washed with a 0.85% w/v NaCl solution to eliminate residual QSMs, and OMs. Each factor was tested at two levels (− 1, 1), resulting in eight distinct experimental scenarios (Table 1S), where − 1 indicates the absence of the compound and + 1 reflects the concentration these compounds observed at glucose depletion under the Glc_0_ + and X_0_- condition of 2^2^ factorial design, previously described in the “2^2^ Factorial Design” section. The + 1 concentrations used were OMs (22.7 mM acetoin plus 22.2 mM 2,3-butanediol), LPs (14.5 µM surfactin plus 14.2 µM bacillomycin D), and CSF (10 µM). For CSF, the concentration used corresponds to that reported by Solomon et al. [87]. Acetoin and 2,3-butanediol were of Sigma-Aldrich reactive grade (Sigma Chemical, St. Louis, MO, USA). Lipopeptides (surfactin and bacillomycin D) were purified from *B. velezensis* 83 culture supernatants as described by Luna-Bulbarela et al. [55], and the CSF (ERGMT sequence) was obtained via chemical synthesis (Genscript Company, USA). Cultures were conducted in 250-mL Erlenmeyer flasks containing 25 mL of glucose-free mineral medium, inoculated at an initial concentration of 5 × 10^9^ cells/mL, and incubated at 30 °C and 200 rpm.

**Table 1:** Impact of OMs, CSF, and LPs on sporulation efficiency. The table shows the analysis of variance and multivariable regression of the 2^3^ factorial design. The impact of carbon overflow metabolites (OMs), competence and sporulation stimulating factor (CSF), and lipopeptides (LPs) or their interactions on sporulation efficiency in *B. velezensis* 83 is present through a linear model. The corresponding data were obtained by shaken flask trials. Values for statistical analysis were normalized to Glc_0_ + /X_0_- condition. The significant factors and interactions are those with a *p* value lower than 0.05. The model uses the following coded levels for each of the factors: − 1, 0, and + 1. These levels denote absence, half the concentration, and concentration displayed in Glc_0_ + /X_0_- condition 1-h post-glucose depletion, respectively

Source	Sum of squares	DF	Mean square	F value	p-value Prob > F	Coefficient estimate
Model	1.49	7	0.21	43.17	< 0.0001	**β**_**0**_** = 0.79**
OMs	0.057	1	0.057	11.59	0.0027	**β**_**1**_** = 0.049**
CSF	0.098	1	0.098	19.91	0.0002	**β**_**2**_** = 0.064**
LPs	0.85	1	0.85	171.4	< 0.0001	**β**_**3**_** = −0.19**
OMs*CSF	0.22	1	0.22	45.26	< 0.0001	**β**_**4**_** = −0.096**
OMs*LPs	0.19	1	0.19	37.70	< 0.0001	**β**_**5**_** = −0.088**
CSF*LPs	0.00554	1	0.00554	1.120	0.3017	**β**_**6**_** = **0.015
OMs*CSF*LPs	0.075	1	0.075	15.22	0.0008	**β**_**7**_** = 0.056**
Residual	0.1	21	0.00494			
Lack of Fit	0.042	1	0.042	13.58	0.0015	
Pure Error	0.062	20	3.09E-03			
Cor Total	1.6	28				
R-Squared	0.935					

Linear Model:

%Eff_Spo_ =  + β_0_ + β_1_OMs + β_2_CSF + β_3_LPs + β_4_OMs*CSF + β_5_OMs*LPs + β_7_OMs*CSF*LPs

%Eff_Spo_ =  + 0.79 + 0.049OMs + 0.064CSF—0.19LPs—0.096OMs*CSF—0.088OMs*LPs + 0.056OMs*CSF*LPs

### Analytical Methods

The determination of cellular concentration and spore detection was performed using cell suspensions derived from bioassays. Direct cell counts of *B. velezensis* 83 was performed using a Neubauer chamber (8100104, Hirschmann, Eberstadt, DE) by bright-field microscopy (Optiphot-2 Nikon, Japan) equipped with a 40 × /0.65 160/0.17 objective. For *Bacillus* spore detection, suspensions of cells resuspended in 0.85% (w/v) saline solution at OD = 0.3 (λ = 600 nm) were placed on microscope slides and subjected to dual staining with malachite green and fuchsin (0.1% w/v). Subsequently, one thousand cells were analyzed per sample by microscopic examination [12, 22]. Cell-free supernatant samples were used to determine the concentrations of glucose, carbon overflow metabolites (acetoin and 2,3-butanediol), and lipopeptides (surfactin and bacillomycin D). Glucose concentration was measured employing the glucose oxidase method by YSI 2700D Select Biochemistry Analyzer. Acetoin and 2,3-butanediol concentrations were analyzed via HPLC (Waters 2695) employing an Aminex HPX-87H 7.8 × 300 mm column (Bio-Rad Laboratories Inc, CA, USA) maintained at 30 °C with a constant flow rate of 0.6 mL/min of 5 mM H_2_SO_4_. Acetoin was detected at 210 nm (Waters 2996 Photodiode Array Detector) and 2,3-butanediol by refractive index (Waters 2414). Standard solutions of known concentrations of both compounds were used as references (Sigma Chemical, St. Louis, MO, USA). Finally, surfactin and bacillomycin concentrations were determined by HPLC using a Zorbax C_8_ 5 µm 4.6 mm × 150 mm column (Agilent Technologies, CA, USA), with a constant flow of a mixture of water and acetonitrile supplemented with 0.1% v/v trifluoroacetic acid (TFA) at 1 mL/min and 30 °C. Lipopeptides were separated using a gradient method as detailed by Luna-Bulbarela et al. [54]. Detection was performed at 200 nm with a 2487 dual channel UV/VIS detector (Waters, Milford, MA, USA). Standard solutions of iturin A and surfactin from *Bacillus subtilis*, both with > 95% purity (Sigma-Aldrich Merck KGaA, Darmstadt, DE), served as controls.

### Sporulation Dynamic Characterization

The study focused on four variables to characterize sporulation dynamics comprehensively: (1) sporulation frequency (Freq_Spo_), (2) sporulation efficiency (Eff_Spo_), (3) half-sporulation time (*t*_1/2 Spo_), and (4) sporulation interval (I_Spo_). Sporulation frequency quantifies the proportion of spores (*n*. spores) relative to the total number of cells analyzed (n. cell = *n*. spores + *n*. vegetative cells) at a specified time, defined by Freq_Spo_ = [*n*. spores / (*n*. spores + *n*. cell)] × 100. Sporulation efficiency denotes the fraction of spore concentration at a specified time ([Spores]_*ti*_) relative to the maximum cell concentration during cultivation ([Cell]_*Max*_), formulated as Eff_Spo_ = [Spores]_*ti*_/[Cell]_*Max*_. Half-sporulation time (*t*_1/2 Spo_) indicates the time required to achieve half of the maximum spore concentration following nutritional limitation. Sporulation interval refers to the duration between the appearance of the first spores (*t*_*1S*_, where Freq_Spo_ ≥ 2%) and the time of maximum spore concentration (*t*_*f*_), expressed as *I*_*Spo*_ = *t*_*f*_ – *t*_*1S*._

### Statistical Analysis

Experiments were conducted in triplicate, and differences between means were analyzed using analysis of variance (ANOVA) and Tukey’s test (*n* = 3, *p* ≤ 0.05). The results from experimental design 2^3^ (detailed in the “2^3^ Factorial Design” section of the “Materials and Methods”) were analyzed through ANOVA, empirical modeling with regression methods, and response surface method (RSM) using Design-Expert V.8 software (Stat-Ease, Inc., Minneapolis, MN).

## Results

### Effect of Cell Concentrations and Glucose Level on Metabolite Synthesis and Sporulation in *B. velezensis* 83

**Fig. 1:**
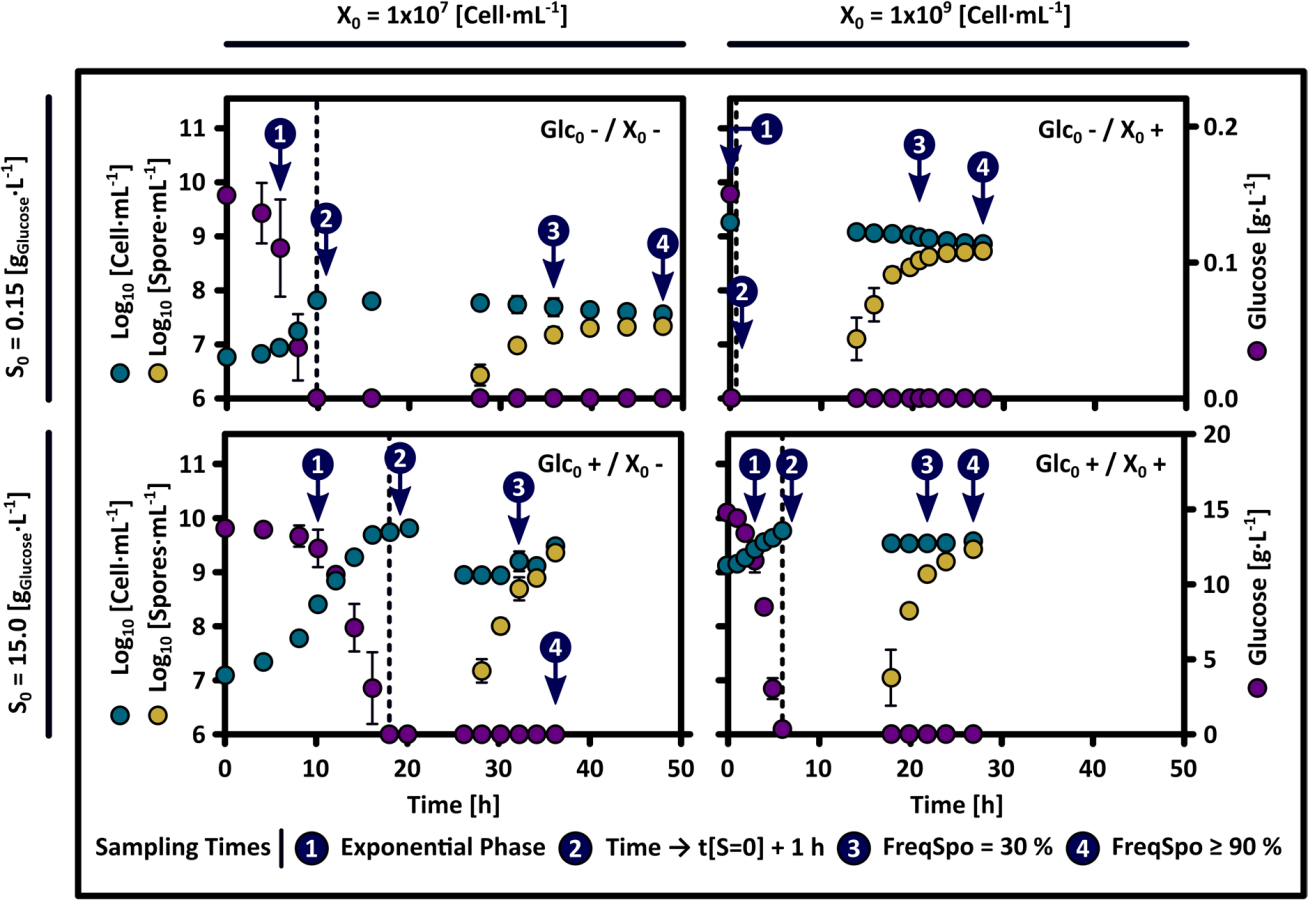
Growth kinetics, sporulation dynamics, and glucose consumption in *B. velezensis* 83 batch cultures. Results from the 2^2^ factorial experimental design assessing the effects of varying initial glucose concentrations (Glc_0_ +  = 15 g/L, Glc_0_- = 0.15 g/L) and cell densities (X_0_ +  = 1 × 10^9^ cells/mL, *X*_0_- = 1 × 10.^7^ cells/mL) on sporulation profile are illustrated in the graphs. Green, yellow, and purple circles are total cell count, spores, and residual glucose levels, respectively. The dashed line shows the time point of glucose depletion (nutritional stress). Numbers in blue circles denote sampling times for quantifying carbon overflow metabolites and lipopeptides at various physiological stages (see Fig. 3)

**Fig. 2:**
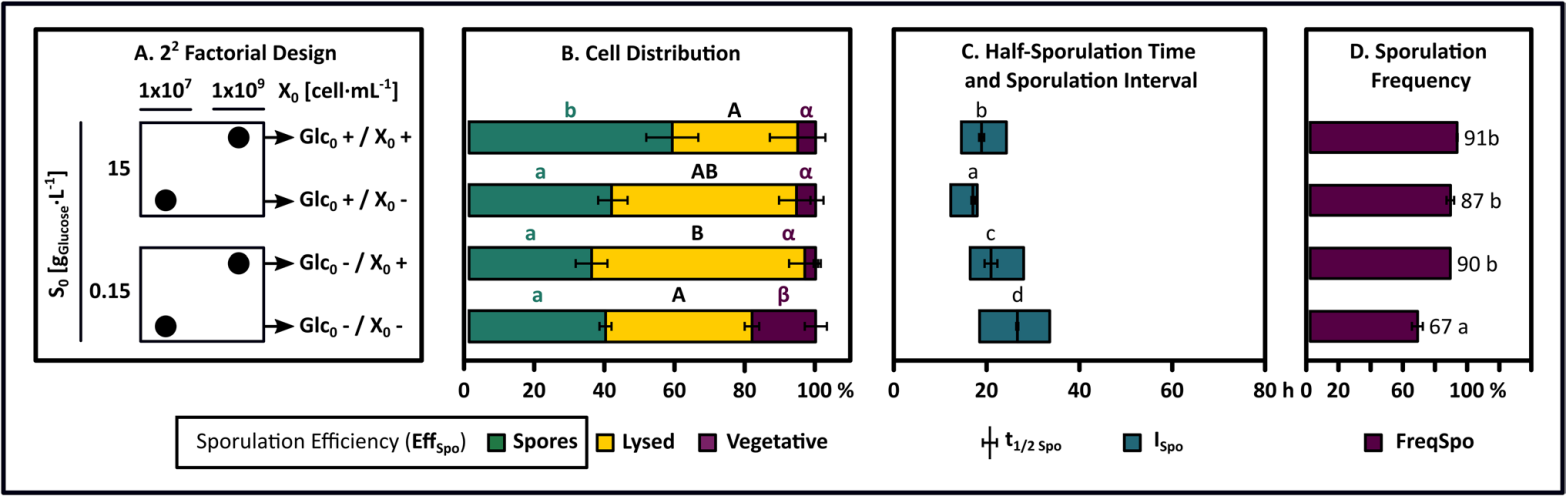
Distribution of subpopulations and sporulation dynamics in *B. velezensis* 83 during batch cultures in stirred tank bioreactor. **A** 2^2^ factorial experimental design to evaluate the effects of varying initial glucose concentrations (Glc_0_ +  = 15 g/L, Glc_0_- = 0.15 g/L) and cell densities (X_0_ +  = 1 × 10^9^ cells/mL, X_0_- = 1 × 10.^7^ cells/mL), on distribution of subpopulations and sporulation dynamics in *B. velezensis* 83. Black circles denote the distinct combinations that were analyzed. **B** Subpopulation distribution at the final evaluated time of each culture. Here, green, yellow, and purple colors show the percentage of cells corresponding to spores, cells lost due to lysis, and vegetative cells compared to the maximum observed total cell concentration, respectively. In this case, the spore percentage also denotes sporulation efficiency (%Eff_Spo_). **C** Half-sporulation time (*t*_1/2 Spo_) and sporulation interval (*I*_Spo_). Initial times are relative and adjusted to the glucose depletion in each culture. **D** Sporulation frequency at the final evaluated time. Different letters indicate significant differences (Tukey, *n* = 3, *p* < 0.05)

**Fig. 3:**
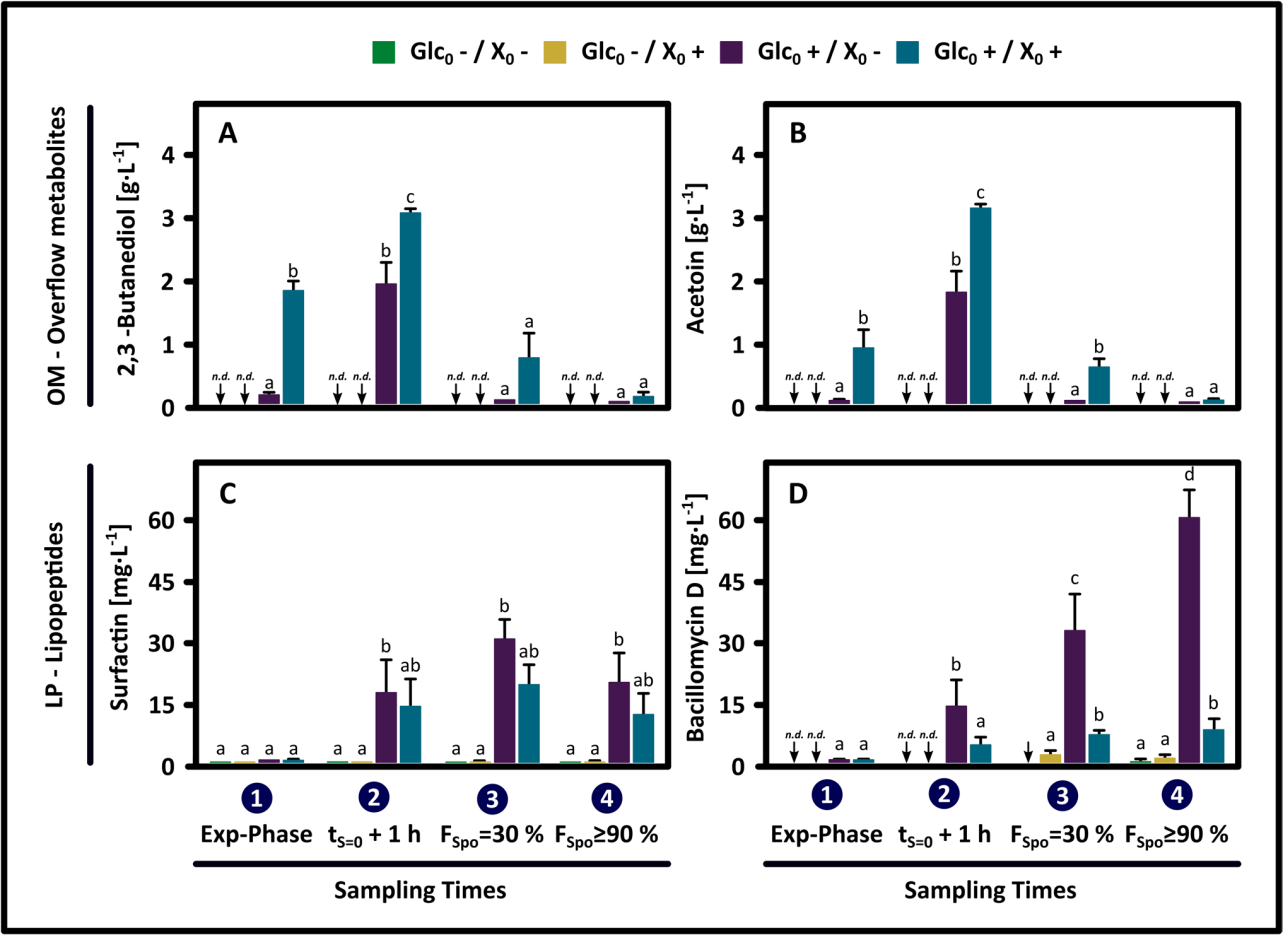
Kinetics of carbon overflow metabolite and lipopeptide production by *B. velezensis* 83 in batch culture. Results derived from a 2^2^ factorial experimental design to evaluate the impact of varying initial glucose concentrations (Glc_0_ +  = 15 g/L, Glc_0_- = 0.15 g/L) and cell concentrations (X_0_ +  = 1 × 10^9^ cells/mL, X_0_- = 1 × 10^7^ cells/mL) on the production of 2,3-butanediol, acetoin, surfactin, and bacillomycin D. Sampling times correspond to distinct physiological stages: (1) Mid-exponential growth phase (Exp-Phase), (2) 1-h post-glucose depletion (*t*_S=0_ + 1), (3) upon reaching 30% sporulation frequency (*F*_Spo_ = 30%), and (4) final sporulation stage (*F*_Spo_ ≥ 90%). The sampling points are shown in blue circles, as in Fig. 1. Statistical significance is denoted by different noncapital letters (Tukey, *n* = 3, *p* < 0.05). ↓n.d. indicates non-detectable levels

Figures 1 and 2 show bacterial growth, sporulation, and residual glucose profiles for the four experimental conditions outlined in the 2^2^ design. Additionally, the concentration of OMs and LPs at four distinct physiological stages of these cultures is shown in Fig. 3. The cultures were analyzed in both the growth phase and the nutrient limitation phase. These phases were temporally distinguished by the point of glucose depletion (t[S → 0]), confirming glucose as the limiting substrate. During the cell growth phase, OMs and surfactin were synthesized, where their accumulation levels were proportional to the initial substrate concentration. The period of nutrient limitation was marked initially by a stationary phase, where bacillomycin D accumulated to its highest levels. After this period, the emergence and subsequent accumulation of spores were observed, coinciding with a reduction in cell concentration caused by cell autolysis and the utilization of the synthesized OMs (Figs. 1 and 3). The analysis of the four conditions revealed that the final distribution of cells (vegetative cells, spores, and cells lost through lysis) and the sporulation profiles, in terms of sporulation efficiency, half-sporulation time, sporulation interval, and frequency sporulation, were predetermined by the initial substrate concentration and the secondary metabolites generated during the growth phase (Fig. 2). Moreover, sporulation consistently occurs after glucose depletion, independent of exogenous nutrient sources (OMs), lipopeptide concentrations, or the initial cell concentration. The following provides a detailed description of the observations collected.

#### OMs Generated During the Growth Phase Are Consumed During the Period of Nutritional Starvation, in Synchronization with the Sporulation Process

For all treatments, although the initial spore formation was detected at different cultivation times, it always occurred between 10 and 18 h after glucose depletion (Fig. 1). Therefore, glucose depletion was the main factor triggering the sporulation activation in *B. velezensis* 83. This observation agreed with prior knowledge, which described morphogenesis as an asynchronous process within a population, where individual cell differentiation needed between 6 and 8 h [66, 83, 85]. Even if the sporulation process was completed between 18 and 34 h for all cultures, the associated lag phase, half-sporulation time, and specific sporulation interval were affected by culture conditions. In Figs. 1 and 2, it was clear that treatments with elevated initial glucose levels (Glc_0_ + /X_0_ + y Glc_0_ + /X_0_-) exhibited a reduced lag phase in sporulation. Furthermore, the half-sporulation times and sporulation intervals for these treatments were shorter as compared to those with low initial glucose levels (Glc_0_-/X_0_ + y Glc_0_-/X_0_-). While the condition with the highest production of overflow metabolites (Glc_0_ + /X_0_ +) presented the highest sporulation efficiency, the condition with the highest lipopeptide yield (Glc_0_ + /X_0_-) had the shortest half-sporulation time and sporulation interval (Figs. 2 and 3). After the depletion of glucose, no additional increase in the total cellular concentration was detected in treatments that yielded large quantities of OMs, despite the gradual consumption of these metabolites during the limitation phase (Fig. 3). Nonetheless, it is plausible that some cells continued to proliferate, but their growth rate was offset by an elevated rate of cell death. From these results, the following hypothesis was proposed: an increased accumulation of secondary metabolites in the culture medium, such as LPs or OMs, enhanced sporulation by improving cellular synchronization or supplying the essential resources for spore formation, without directly triggering its activation. As expected, the maximum spore concentration was significantly influenced by the initial substrate concentration (Fig. 1); however, high initial concentration of both cells and glucose (Glc_0_ + /X_0_ +), with initial glucose level theoretically adequate to produce the same initial cell number, did not result in a significant increase in the final concentration of total cells (X_Max_ = 5 × 10⁹ cells/mL), nor did it affect the sporulation frequency relative to the control condition (Glc_0_ + /X_0_-). Nonetheless, the Glc_0_ + /X_0_ + condition did result in a 50% increase in sporulation efficiency (from 0.4 to 0.6 spores/cell) relative to Glc_0_ + /X_0_- (Figs. 1 and 2). However, the concentration of surfactin and bacillomycin produced during the Glc_0_ + /X_0_- condition were 60% and 650% higher, respectively, than those produced by the Glc_0_ + /X_0_ + condition (Fig. 3). Since the synthesis of these lipopeptides is growth associated, these differences can be attributed to consistent variations in specific growth rate (µ), which were 0.4 h⁻^1^ for Glc_0_ + /X_0_- and 0.29 h⁻^1^ for Glc_0_ + /X_0_ + . Genes involved in sporulation are transcribed when a high level of phosphorylated Spo0A is achieved [21]. Low specific growth rates contribute to the accumulation of phosphorylated Spo0A, thereby enhancing the probability of sporulation [68]. This effect is attributed to the prolonged exposure to quorum-sensing molecules and the increased accumulation of phosphorelay proteins [68]. Along with the contribution of a higher generation OMs and their subsequent consumption, this might explain why Glc_0_ + /X_0_ + exhibited significantly higher sporulation efficiency than the Glc_0_ + /X_0_- condition, even with the same maximum total cell concentration.

Under a condition of glucose starvation, the process of assimilating alternative nutrients (e.g., PGA, succinate, fumarate, peptidoglycan residues, acetate, acetoin, 2,3-butanediol) previously released into the culture medium is triggered [7, 12, 13, 29, 57, 71, 79, 98]. In nutritional terms, the aforementioned metabolites could significantly support the cellular economy and spore formation by providing carbon skeletons and energy. Throughout the performed cultures, acetoin and 2,3-butanediol (OMs) were accumulated extensively in the culture medium of *B. velezensis* 83. In the Glc_0_ + , X_0_ − condition, an apparent acetoin plus 2,3-butanediol yield of 0.18 g_P_/g_S_ was observed. Earlier research indicated that the biomass yield in *B. velezensis* 83 had an inverse correlation with glucose availability, where elevated glucose levels were associated with a predominant diversion of carbon towards the synthesis of OMs such as acetoin, 2,3-butanediol, and γ-PGA [12, 13]. As reported in the literature, the metabolism of acetoin and 2,3-butanediol produces acetyl-CoA via the acetoin dehydrogenase enzyme system (encoded within the *acoABCL* operon) facilitating the reentry of this carbon into central metabolism through key pathways such as the tricarboxylic acid (TCA) cycle and lipid biosynthesis [73]. In fact, the proteins involved in the TCA cycle are significantly upregulated during the stationary phase (glucose starvation scenario), since this pathway plays an essential role in energy production under these conditions and during the sporulation process [58, 71]. Chromosome translocation requires the hydrolysis of considerable amounts of ATP by the mother cell SpoIIIE subcomplex [53]. Furthermore, acetyl-CoA is indispensable for fatty acid biosynthesis, which is crucial for the formation of the forespore membrane. Although γ-PGA concentration was not determined in the present cultures, previous comparable experiments have indicated that under the Glc_0_ + /X_0_- condition, a peak concentration of 1.4 g/L of this biopolymer is reached, which subsequently decreases gradually in alignment with sporulation [5, 12]. This indicates that cultures with a high initial glucose concentration are characterized by starting the nutrient limitation and sporulation phase with elevated levels of acetoin, 2,3-BD, as illustrated in Fig. 3, and possibly with the presence of γ-PGA. In addition to LPs, OMs and PGA, other metabolites, such as peptidoglycan (PG) fragments, could impact sporulation profiles. During the growth of Gram-positive bacteria, PG fragments are released into the culture medium and later recycled, improving survival in the stationary phase [7, 79]. In *B. subtilis*, around 10% of the N-acetylmuramic acid (a sugar derived from PG) produced per generation is recycled [7]. As these metabolites are growth-associated products, it is reasonable to propose that those treatments with high initial glucose levels (Glc_0_ + /X_0_ + and Glc_0_ + /X_0_-), characterized by greater biomass generation, display higher concentrations of these compounds relative to other conditions (Glc_0_-/X_0_ + and Glc_0_-/X_0_-) at the onset of nutrient limitation.

#### The Cell Concentration Did Not Influence the Efficiency of Sporulation, But It Possibly Dictated the Amount of Nutrients Obtained by Cell Autolysis

The scenarios involving low initial glucose availability under different initial cell concentrations (Glc_0_-/X_0_- and Glc_0_-/X_0_ +) enabled the evaluation of how cell concentration influences sporulation profiles. Under these two conditions, where the concentrations of lipopeptides, acetoin, and 2,3-BD can be considered negligible, it is noteworthy that sporulation still occurs, with efficiency comparable to that of the control scenario (Glc_0_+/X_0_-). During all experimental conditions outlined in the 2^2^ design, the emergence of the first spores takes place hours after both glucose starvation and the accumulation of the two lipopeptides (surfactin and bacillomycin), meaning that the effect of sporulation induction through lipopeptides cannot be excluded. The conditions with low initial glucose concentration (Glc_0_-/X_0_- and Glc_0_-/X_0_ +) suggest that high concentrations of these molecules are not necessary to induce sporulation (Figs. 1 and 2). Furthermore, the comparison of sporulation profiles between Glc_0_-/X_0_- and Glc_0_-/X_0_ + scenarios demonstrated that higher initial cell concentrations facilitated cellular synchronization, resulting in shorter half-sporulation time and sporulation interval, and a higher frequency of sporulation. From a subpopulation balance perspective, the sporulation frequency alone is a parameter insufficient to compare both scenarios, as it does not consider cells lost through cell lysis. In terms of sporulation efficiency, both treatments did not show significant differences, which leads to discussing previous approaches on the effect of cell concentration on sporulation. At high initial cell concentration (Glc_0_-/X_0_ +), a higher level of cell autolysis, at the expense of the vegetative cell fraction, provided more nutrients compared to the Glc_0_-/X_0_- scenario (Fig. 2). Later, to determine whether the lysis products alone could supply the necessary elements to complete sporulation, the dynamics of sporulation were analyzed for cells resuspended solely in saline solution (0.9% w/v NaCl) at an initial concentration of 5 × 10⁹ cells/mL. In contrast to the comparable condition of low initial glucose (Glc_0_-/X_0_ +), these cells lacked any external sources of carbon, nitrogen, phosphorus, or other micronutrients. The findings revealed that even in the total absence of external nutrients, *B. velezensis* 83 remained capable of sporulating with a sporulation frequency of 76%, although the efficiency was as low as 22%. The high percentage of lysed cells (71%) indicated that the observed sporulation was predominantly sustained by cell autolysis. Therefore, in *B. velezensis* 83, the development of the cannibalistic phenotype, together with cell autolysis due to normal cellular senescence and death, represent mechanisms that are critical for nutrient recycling and ensuring the availability of resources necessary for sporulation.

### Influence of OMs, LPs, CSF, and Their Interactions on the Sporulation Profiles

**Fig. 4:**
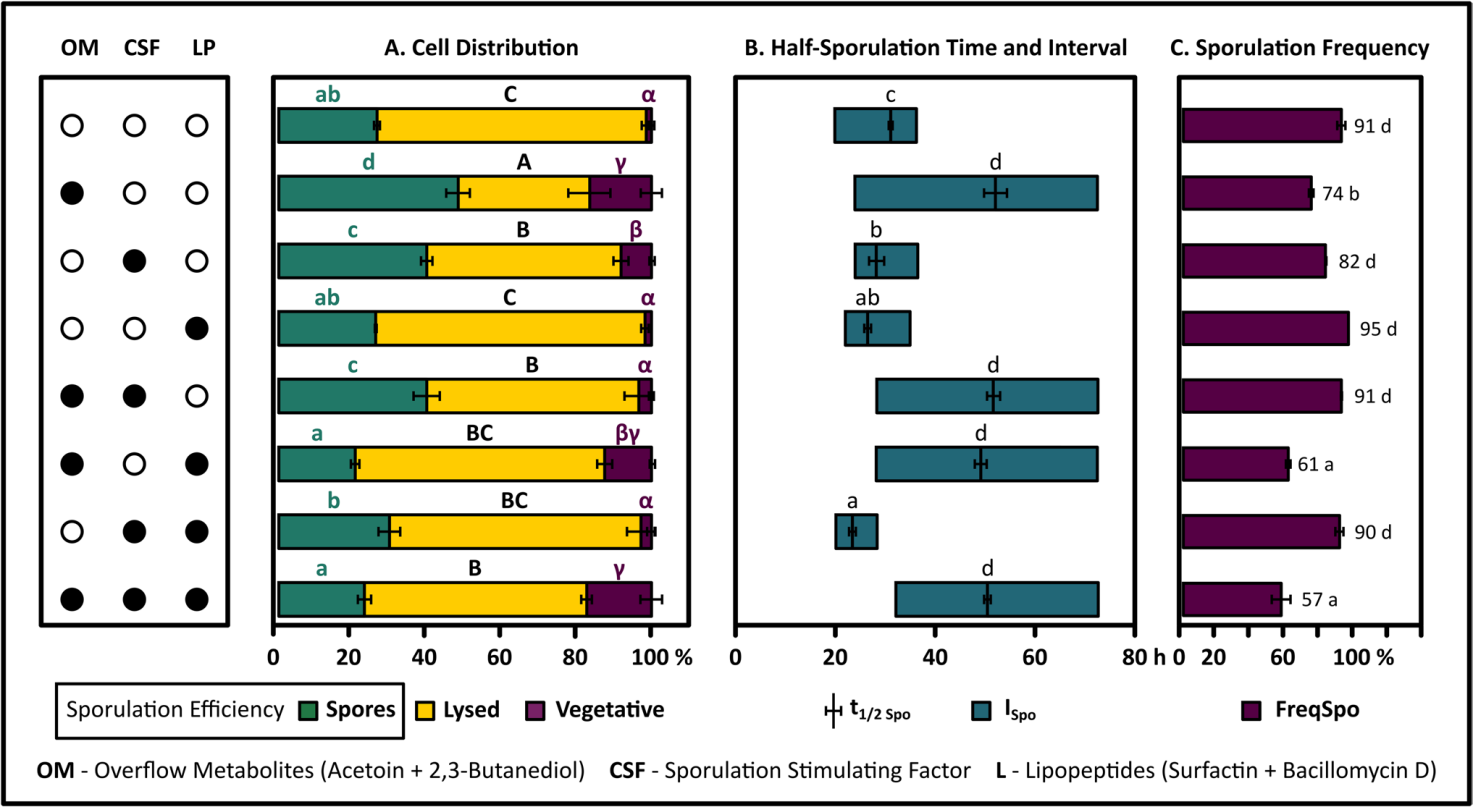
Impact of exogenous addition of quorum-sensing and overflow metabolites on subpopulation distribution and sporulation dynamics in *B. velezensis* 83. The data presented were derived from experiments conducted in shaked flasks with glucose-free mineral medium, starting with an initial cell concentration of 5 × 10^9^ vegetative cells/mL. The treatments shown on the left use combinations of black and white circles to show presence or absence of the following factors: Overflow metabolites (OMs, 22.7 mM acetoin plus 22.2 mM 2,3-butanediol), competence and sporulation stimulating factor (CSF, 10 µM), Lipopeptides (LPs, 14.5 µM surfactin plus 14.2 µM bacillomycin D). The control condition is represented by three empty circles. **A** Subpopulation distribution at the final evaluated time of each culture. Here, green, yellow, and purple colors show the percentage of cells corresponding to spores, cells lost due to lysis, and vegetative cells compared to the maximum total cell concentration (5 × 10.^9^ cell/mL), respectively. The spore percentage also denotes sporulation efficiency (%Eff_Spo_). **B** Half-sporulation time (*t*_1/2 Spo_) and sporulation interval (*I*_Spo_). **C** Sporulation frequency at the final evaluated time. Different letters indicate significant differences (Tukey, *n* = 3, *p* < 0.05)

**Fig. 5:**
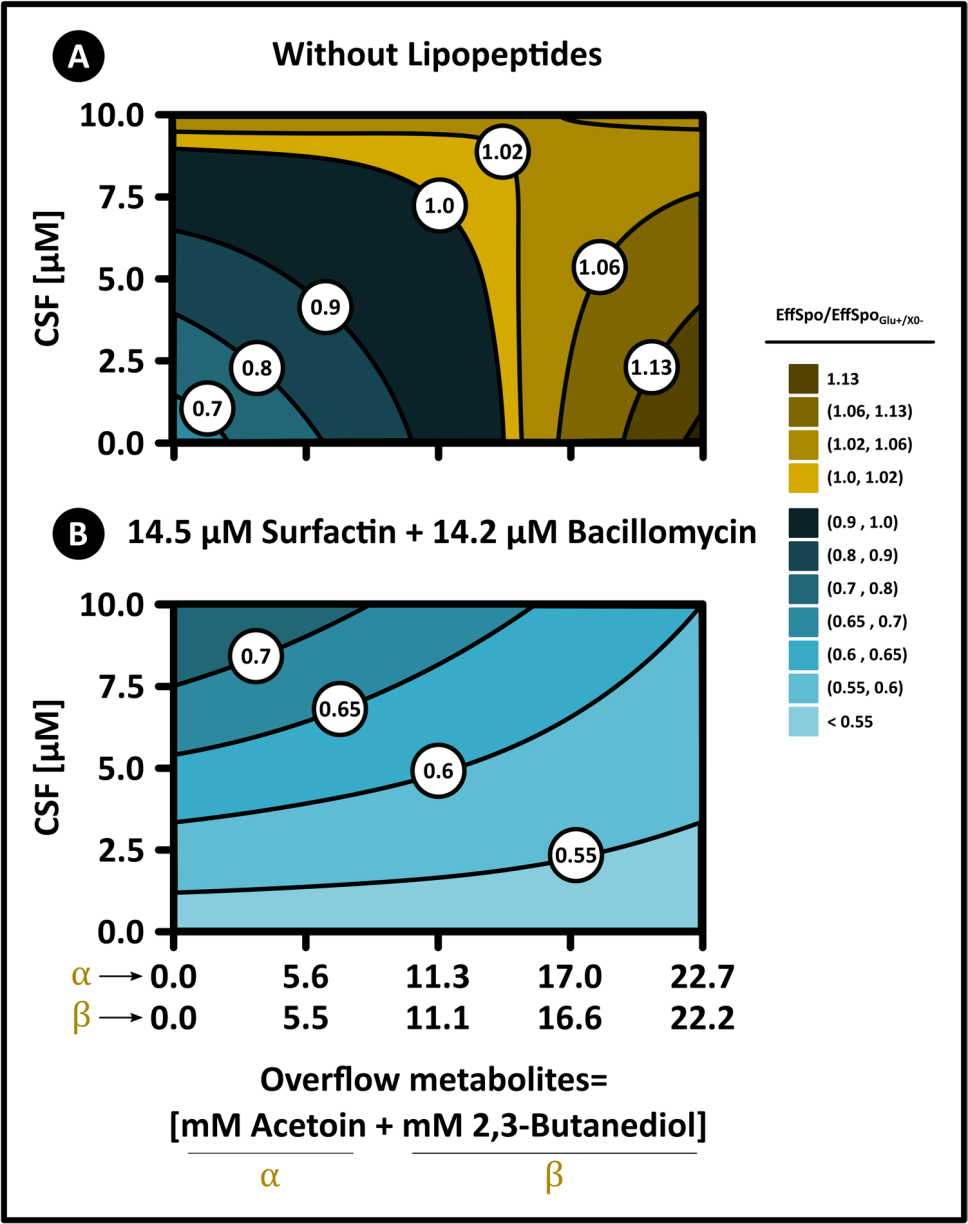
Two-dimensional response surface analysis. The plot shows the interactions between quorum-sensing metabolites and carbon overflow metabolites on sporulation efficiency in *B. velezensis* 83. The indicated concentration of carbon overflow metabolites represents the combined levels of acetoin (α) and 2,3-butanediol (β). The color gradient indicates normalized sporulation efficiency relative to that observed in the Glc_0_ + /X_0_- culture

In order to confirm the aforementioned hypothesis and to evaluate the impact of overflow metabolites (OMs, acetoin + 2,3-butanediol), lipopeptides (LPs, surfactin + bacillomycin D), and CSF (a QSM) on the sporulation profiles of *B. velezensis* 83, an experimental design comprising 2^3^ combinations was established, imitating specific in vitro scenarios of nutrient limitation that are comparable to the previously mentioned Glc_0_ + /X_0_- condition. The Glc_0_ + /X_0_- condition served as a reference due to its short sporulation phase, which suggested a high level of cellular synchronization making it easier to identify possible effects of each factor. Furthermore, it was selected as a representative scenario of a spore production process. In each condition, the corresponding mass of metabolites were added to replicate the concentration observed in the Glc_0_ + /X_0_- condition at the point of glucose depletion (sample t_S0_ + 1 h, Fig. 3). Figure 4 shows the distribution of subpopulations, sporulation efficiency (%Eff_Spo_), half-sporulation time (*t*_1/2-Spo_), and sporulation interval (*I*_Spo_) for the eight experimental conditions detailed in the 2^3^ design (Table 1S). According to a multiple comparisons test (Tukey, *p* < 0.05), significant differences were found among the treatments concerning all response variables (Fig. 4). Subsequently, the main effects and interactions of these three factors on sporulation efficiency were evaluated by ANOVA, multivariable linear regression, and response surface method, as described in Table 1 and Fig. 5. For the latter, the values for statistical analysis were normalized with respect to the Glc_0_ + /X_0_- condition. The principal findings are described below.

The analysis of variance demonstrated that each of the three factors investigated had a significant individual impact on sporulation efficiency (OMs, CSFs, and LPs with *p*-values of 0.0027, 0.0002, and < 0.0001, respectively), in addition to the significant two-factor interactions (OMs*CSF and OMs*LPs with *p*-values of < 0.0001 and < 0.0001, respectively) and the three-factor interaction (OMs*CSF*LPs with a *p*-value of < 0.0008). The CSF*LP interaction was the only one whose contribution was not significant (*p*-value = 0.3) (Table 1). The multivariable linear regression model, with a high coefficient of determination (*R*^2^ = 0.93, Table 1), revealed that carbon overflow metabolites (OMs, *β*₁ = 0.049) and the competence and sporulation stimulating factor (CSF, *β*₂ = 0.064) positively influenced sporulation efficiency, while lipopeptides negatively affected it (LPs, *β*₃ =  − 0.19). Conversely, the OMs*CSF and OMs*LPs interactions had a negative impact (*β*₄ =  − 0.096 and *β*₅ =  − 0.088, respectively), while the three-factor interaction, OMs*CSFs*LPs, showed a positive effect (*β*₇ = 0.056). Even though the main effect of the factors was significant, the statistical validation of the model through the lack of fit test showed that a significant curvature exists at the center of the experimental domain (Table 1), implying that the interaction among the factors is more complex and nonlinear. Nevertheless, the factorial experiment estimations were adequate (*R*^2^ = 0.935) to analyze and discuss the main effects of the factors on sporulation efficiency if no optimization of the responses is intended. Other QSMs also displayed non-linear responses in their dose–response curves, such as the sigmoidal relationship observed between the transcription of the surfactin synthetase gene *srfA* and the extracellular concentration of the pheromone ComX [17]. On the other hand, ANOVA analysis of the linear model showed that OMs are the only factors influencing the timing and interval of sporulation, but with correlations below 0.78 (Table 1S). As a result, only the Tukey test was used to examine significant differences between treatments in relation to these two variables.

#### LPs and CSF Displayed Opposite Effects on Sporulation Efficiency, But Both Consistently Increased Cellular Synchronization

Lipopeptides were the factor that had the most significant (negative) effect on sporulation efficiency. In terms of sporulation frequency, treatments with addition of lipopeptides did not show differences compared to the control, except for the OMs*LPs interaction, which had a negative effect (Fig. 4). Due to their surfactant properties, surfactin and bacillomycin can permeabilize and damage cell membranes in bacteria and fungi, respectively [74, 101]. The fraction of lysed cells observed in the lipopeptide treatments was similar to that in the negative control (Fig. 4), suggesting that the lipopeptides did not have a detergent effect. Although lipopeptides caused a negative effect on sporulation efficiency, they also negatively influenced the half-sporulation time and sporulation interval, supporting the hypothesis that they promote cellular synchronization. Nevertheless, this effect can be fully counteracted by interaction with OMs (Fig. 4).

Within the scope of industrial spore production, the observations described previously are relevant since the sporulation interval can act as an indirect metric for assessing spore quality. Accelerated sporulation processes led to the accumulation of spores with a high germination rate, a highly valued characteristic [66]. Consequently, the effects of CSF could have significant implications, enhancing spore quality indirectly via the acceleration of the sporulation process. CSF treatment had a positive impact on both sporulation efficiency and cellular synchronization (shorter half-sporulation time and sporulation interval) with respect to control (Fig. 4). The signaling peptide CSF promotes sporulation through its interaction with Rap-type phosphatase, inhibiting their activity and thereby avoiding the dephosphorylation of intermediates that trigger Spo0A activation [45]. Therefore, the results were consistent with what was expected. In a different comparison, the exogenous supplementation of CSF at a concentration of 10 µM recovered the sporulation profile of the Glc_0_ + /X_0_- culture, in both efficiency and frequency of sporulation, showing that this signaling peptide was essential for enhancing the number of cells that exceeded the Spo0A-P threshold (Fig. 4 and Table 1S). However, in the same comparison, CSF by itself did not recover the half-sporulation time and sporulation interval (Fig. 4 and Table 1S). The two-factor interaction, CSF*LPs, provided evidence in this regard. Even though the CSF*LPs interaction (*β*_6_ = 0.015, *p*-value = 0.3017) did not significantly affect sporulation efficiency with respect to negative control (Fig. 4), it fully restored the sporulation profile of the Glc_0_ + /X_0_- culture in terms of *I*_Spo_, *t*_1/2 Spo_, and Freq_Spo_. In other words, it replicated the individual contributions of both CSF and LPs, reaffirming the potential positive impact of LPs on synchronization.

#### The OMs Positively Influenced Sporulation Efficiency But Exerted a Robust Negative Effect on Cellular Synchronization

Aligned with the hypothesis presented in the “OMs Generated During the Growth Phase Are Consumed During the Period of Nutritional Starvation, in Synchronization with the Sporulation Process” section, treatment with carbon overflow metabolites (acetoin + 2,3-BD) confirmed their positive effect on sporulation efficiency, significantly changing the distribution of *B. velezensis* 83 subpopulations. The observed distribution displayed a reduced proportion of lysed cells and an increased proportion of both spores and residual vegetative cells (Fig. 4). Even with a reduced sporulation frequency, the absolute total amount of spores generated in the OMs treatment was higher than in the control (up to 1.8-fold increase), highlighting the nutritional importance of these metabolites. *B. velezensis* 83 consumed all the supplied acetoin and 2,3-BD during the nutrient limitation phase, in synchrony with the sporulation process. Combined with nutrients obtained via cell autolysis, the energy provided by these compounds sustained the energy demand necessary to keep viability and promote cellular differentiation. However, the OMs induced an unintended effect on the sporulation profile, consistently prolonging both the half-sporulation time and sporulation interval by 2.6 to 2.9 times, which is a clear sign of cellular desynchronization. The robust positive effect of OMs on these two variables was consistently observed in all treatments involving OMs, regardless of the influence exerted by other factors (CSF or LPs). The multivariable linear regression analysis supports the positive effect of OMs on both variables, although with a low level of correlation (*R*^2^ of 0.84 and 0.78, respectively) (Tables 1–3S).

These findings suggest that the interactions between the different factors employed in the in vitro nutrient limitation scenarios (OMs, CSF, and LPs) are complex. Treatment with OMs as the sole factor increased sporulation efficiency by 20% compared to the Glc_0_ + /X_0_- culture, supporting the idea that interactions with other excreted components, such as lipopeptides, could negatively affect efficiency (Fig. 4 and Fig. 5). This aligns with the multivariable linear model and response surface analysis, which predicted that the interaction between OMs and QSMs (LPs or CSF) displays an antagonistic interaction, affecting sporulation efficiency (Fig. 4 and Fig. 5). This was consistent with the double interactions treatments (OMs*CSF and OMs*LPs), but in contrast to the results observed with the OMs addition treatment. The sporulation efficiency resulting from the three-factor interaction treatment (OMs*CSF*LPs) was equivalent to the negative control, reinforcing the presence of antagonistic interactions among the factors (Fig. 4 and Table 1S). In fact, this treatment achieved only 58% of the sporulation efficiency displayed in the Glc_0_ + /X_0_- culture (Fig. 5). On the other hand, the three-factor interaction treatment (OMs*CSF*LPs) had a positive impact (increase) on half-sporulation time and sporulation interval, inducing cellular desynchronization equivalent to the individual OMs effect. Similarly, a substantial increase in the proportion of remaining vegetative cells was detected in OMs*CSF*LPs treatment, which caused a decrease in sporulation frequency. Comparing the sporulation profile of the negative control with the Glc_0_ + , X_0_- condition, the latter showed a shorter half-sporulation time and sporulation interval, indicating that there may be other molecules involved in the cellular synchronization of *B. velezensis* 83 in addition to LPs and CSF. The in vitro limitation control scenario is lacking other QSMs, including other Phr mature peptides (PhrF, PhrK, and PhrA mature peptides), which could have a positive effect on the *B. velezensis* 83 sporulation.

## Discussion

### Significance of Improved Bioprocess Development for Producing Wild-Type *Bacillus* Spores via Exogenous Induction Strategies

Spore-forming Bacilli, such as *B. subtilis* and *B. velezensis* (also known as *B. amyloliquefaciens*), are bacteria commonly used as a probiotic due to their antimicrobial properties [35, 43, 64], and multiple health and growth promoting effects [4, 6, 9]. In agriculture, biocontrol products based on plant growth-promoting rhizobacteria (PGPR) such as *B. subtilis*, *B. velezensis*, and *B. amyloliquefaciens* are among the most widely commercialized for Integrated Pest Management (IPM) strategies [69]. In addition, these bacteria were also suggested as prospective probiotics for use in the aquaculture of multiple fish species, including *Labeo rohita* [38], *Micropterus salmoides* [9], *Oreochromis niloticus* [43], and *Dicentrarchus labrax* [64], among others. The application of *Bacillus*-based probiotics supports microbiota balance and enhances nutrient absorption in monogastric animals [4]. *B. amyloliquefaciens* demonstrated therapeutic properties, reducing the pro-inflammatory response in induced colitis animals [30] and preventing biofilm establishment by the pathogenic bacterium *Proteus mirabilis* [2]. Moreover, their application as agents in feed additive production has been suggested [4, 60]. The extensive applications of these microorganisms are manifested in the growing demand for *Bacillus*-based probiotic formulations, emphasizing the necessity for optimized bioprocesses for spore production. However, the application of these microorganisms is restricted to wild strains. Although sporulation in *B. subtilis* is one the most extensively studied models of cellular differentiation in bacteria [82], the inability to employ improved mutant strains presents a significant obstacle for the development of bioprocesses for spore-based probiotics production, as the determinants of sporulation result in intricate control of genetic circuits. Added to the above, like other non-domesticated strains, *B. velezensis* 83 shows resistance to genetic transformation, a trait that, while advantageous for bioformulation stability, also limits the use of molecular techniques for research purposes [63, 89, 99]. This work demonstrates the influence of the metabolites produced during the growth phase of *Bacillus velezensis* 83 on the sporulation kinetics and subpopulation distribution after glucose depletion. This is particularly important in order to improve spore productivity of probiotic industrial processes restricted to wild-type *Bacillus* spp. strains. Previous reports showed the important role of overflow metabolites on *Bacillus* sporulation [3, 29, 51, 52, 98]. On the other hand, it has been shown that biomass (Y_x/s_) and overflow metabolite (Y_P/S_) yield in *B. velezensis* 83 could be modulated by regulating the glucose flow rate in continuous cultures [12, 13]. Increasing glucose flow rates decreased biomass yield and increased overflow metabolite yield in a nonlinear relationship. Fed-batch cultivations could be a valuable approach to identify the optimal glucose feed rate for modifying *Y*_X/S_ and *Y*_P/S_, thereby optimizing spore productivity and efficiency in *B. velezensis* 83 cultures.

### The Sporulation Process, a Biological Pathway Triggered by Nutritional Limitation, But Which Paradoxically Requires Significant Energy Supply

Sporulation initiation is controlled by the response regulator Spo0A, which becomes active through a phosphorylation cascade started by sensor kinases KinA-E, in response to nutrient limitation, or by quorum-sensing signals that have exceeded an activation concentration threshold [32, 85]. Paradoxically, even though spore formation occurs during nutrient limitation, it is an energy-demanding process requiring both genome duplication and the precise synthesis of peptidoglycan and coat proteins within a strictly controlled morphogenesis [61, 92]. In conditions of nutrient limitation and during the sporulation phase, the use of external energy sources (e.g., OMs), internal sources and the recycling of resources from cell autolysis (through cannibalism or normal senescence and death of cells), become important for increasing the ecological fitness of *Bacillus* populations [80, 92].

Regarding the internal resources used for spore assembly (e.g., membrane, peptidoglycan, ribosomes), these are obtained through de novo synthesis, and redistribution and/or remodeling of pre-existing materials, within a sophisticated intercellular metabolic interaction between the mother cell and the forespore [31, 53, 62, 70, 81, 93]. After polar septation, the initial stages of spore formation advance with chromosome translocation mediated by ATP-dependent dsDNA translocase SpoIIIE, and the engulfment of the membrane is assisted by proteins such as the membrane fission protein FisB [16, 39]. Engulfment leads to the formation of a double-membrane structure that defines the forespore [19, 39]. Peptidoglycan remodeling (degradation and biosynthesis) is also involved in this step to enable the distribution of the synthesized membrane during the process [53, 62]. On the other hand, forespore ribosomes are derived from the mother cell, obtained via a translocation mechanism dependent on peptidoglycan remodeling, which occurs temporally after chromosome translocation [31]. Similarly, small molecules that are useful as metabolic building blocks (e.g., arginine) are intercellularly transported from the mother cell to the forespore [81, 93].

It is important to highlight that the negative control used in the 2^3^ experimental design involved vegetative *Bacillus* cells resuspended in a mineral medium without glucose, at a high initial cellular concentration. This means that, excluding the carbon released by lysis and/or internal sources, these cells lack any other accessible carbon source. Indeed, together with the treatments that involved lipopeptide addition, the negative control showed the greatest percentage of cell lysis. Compared to the Glc_0_ + , X_0_ + condition, in this control culture, the concentration of QSMs (CSF and LPs) and OMs is minimal, which resulted in lower sporulation efficiency. These cells must invest resources in the synthesis of proteins vital for signal transduction, including Phr pro-peptides and the proteases involved in their maturation. Nonetheless, the efficiency was significant (0.25 spore/cell). If sporulation activation were dependent on a high concentration of CSF and/or lipopeptides, this level could not have been reached. The activation of sensor kinases KinA-E, linked to nutritional stress, is likely sufficient to induce the phenomenon under these conditions [18, 94]. Considering that the required concentration of the ComX pheromone to elicit a response is low (10 nM for full *srfA* expression, [17]), it can be hypothesized that the concentration achieved in the negative control was sufficient to activate sporulation. The presence of ComX in the extracellular environment plays a key role in triggering the phosphorylation of the ComA regulator, which in turn activates the transcription of genes for surfactin and/or bacillomycin production, thereby affecting sporulation. This QSM is important because *Bacillus subtilis* mutants lacking the ComX pheromone (∆comQ) exhibit only 10% of the sporulation frequency observed in the parental strain [59]. Nevertheless, to properly evaluate the possible influence of ComX on sporulation efficiency in *B. velezensis* 83, it would be necessary to perform assays with synthetic pheromone supplementation.

Similarly to sporulation, the QS process through signaling peptides, including ComX pheromones or Phr mature peptides in *Bacillus* spp., require more energy in terms of ATP and amino acid consumption than other systems, such as N-acyl homoserine lactones in Gram-negative organisms [17, 37]. To compensate for the energetic cost of its production, *Bacillus* spp. developed a highly sensitive QS system, as seen with ComX [17]. Considering that sporulation was elevated during the experiments with low initial glucose concentrations, including the Glc_0_-,X_0_- condition (design 2^2^, Fig. 2) and the negative control (design 2^3^, Fig. 4), the accumulation of ComX molecules at such a level that is sufficient to exceed the activation threshold of the Spo0A phosphorylation pathway in these scenarios is likely to occur. The differences in the sporulation lag phase between the cultures could be attributed to the time required by the cells to accumulate significant levels of the mentioned elements, depending on the nutrients available at the onset of nutritional limitation. With low initial substrate concentrations, cells would initially rely on internal sources and subsequently on nutrients recycled through cell autolysis. Previous evidence suggests that internal cellular sources could contribute significantly [25, 33, 40, 71]. For example, the adaptation of *Bacillus* to glucose starvation involves a complex reconfiguration of cellular metabolism, where a substantial portion of the proteome undergoes degradation via mechanisms like Clp-dependent proteolysis, which down-regulates several proteins involved in central metabolic pathways and facilitates nutrient recycling [25, 40, 71]. Moreover, the expression levels of approximately 3000 genes are altered in *Bacillus* [40]. It is suggested that these non-growing cells are capable of degrading branched-chain fatty acids into pyruvate and succinate, via the methylcitrate pathway [40]. These results align with transcriptome analyses of *B. velezensis* 83, where cells at high concentrations repressed genes involved in fatty acid synthesis and overexpressed those related to their degradation [54], phenotypically corresponding with the reduction in cell size observed during the exponential phase.

For its part, the phenomenon of cell autolysis plays a key role in recycling high-cost nutrients. Spo0A-active cells exhibit cannibalism through the spore killing factor SkfA and the toxin SdpC, which cause lysis in non-sporulating cells, thereby releasing resources into the environment [80], including amino acids such as lysine. This phenomenon leads to the loss of nearly two-thirds of the cells, serving as a mechanism to recycle nutrients and delay sporulation when nutrients are limited [26, 27]. The availability of nutrients resulting from cellular lysis influences the thermoresistance and quality of future spores. The synthesis of dipicolinic acid, a distinctive component of spores that provides thermoresistance, is associated with cell lysis and the liberation of lysine [20]. Under conditions of non-limiting glucose, aspartokinase II LysC starts the lysine biosynthesis pathway but is subject to feedback inhibition by lysine [36]. Upon glucose depletion, LysC is degraded through Clp-mediated proteolysis [25]. In such conditions, aspartokinase I DapG assumes the role of LysC since it is unaffected by feedback inhibition. In a scenario where lysine is released due to cannibalism, DapG ensures the required pool of 4-phospho-L-aspartate (its product) for the synthesis of dipicolinic acid, diaminopimelic acid (a component of peptidoglycan), and lysine needed by the forming spore [20]. This shows the existing and relevant balance between the rate of lysis and the efficiency of sporulation.

### Carbon Overflow Metabolites as Potential Instruments for Enhancing Sporulation Efficiency in *Bacillus velezensis*

Carbon overflow metabolites have traditionally been viewed as unwanted by-products during recombinant protein production. Nevertheless, these metabolites are now recognized for their potential applications in the biocontrol industry and phytostimulation. This work highlights their relevance in sporulation because the metabolites positively affect cellular differentiation, making them promising additives for bioprocesses dedicated to spore production. During the growth phase, acetoin, 2,3-butanediol (2,3-BD) and their stereoisomers are synthesized from pyruvate through the action of acetolactate synthase (AlsS) and acetolactate decarboxylase (AlsD, [46, 73]). Their production increases with elevated specific glucose uptake rates [13]. However, the acetoin dehydrogenase complex *acoABCL*, that is crucial for OM metabolism, is negatively regulated by catabolite repression through the transcriptional regulator CcpA, until nutrient limitation is reached. Once glucose is depleted, the transcriptional activator AcoR and the sigma factor of RNA polymerase SigL facilitate the use of these OMs [3, 11], coinciding with the activation of the sporulation pathway mediated by the phosphorelay response regulator Spo0A-P. In line with the previous findings, the current results demonstrate that under high glucose concentration conditions, these metabolites are accumulated during the growth phase and later consumed in synchronization with spore production [12, 13]. By-products like OMs significantly affected sporulation profiles by decreasing cell lysis and thereby enhancing sporulation efficiency, leading to an increased total number of final spores. Raising the initial glucose concentration would result in inefficient substrate utilization and would be a suboptimal approach for improving the spore concentration by biomass generation, but incorporating the OMs at critical stages could represent a potential strategy, if it is economically feasible. According to the data previously obtained, acetoin production can be manipulated by the initial substrate concentration or the feeding rate of glucose [13].

Even if the OMs positively influence sporulation efficiency, the possible negative effects of OMs on sporulation should also be considered, particularly their considerable negative influence on cellular synchronization and toxicity. The spores generated under conditions that extend the sporulation period, such as OM treatments, may lead to the formation of a highly heterogeneous spore subpopulation in terms of germinative capacity, which is undesirable [66]. In addition to this previously mentioned limitation, precautions must be considered. A high level of acetoin can negatively affect cell growth in Gram-positive bacteria such as *Lactococcus lactis* and *Bacillus* spp. Acetoin damages DNA and proteins through its keto group and triggers reprogramming of lipid metabolism-related proteins, which leads to a later restructuring of membrane lipids profile [8]. In addition to its nutritional contribution, future investigations into sporulation should evaluate the potential toxic impacts of acetoin on membrane homeostasis, which could be intensified by other factors, including the membrane active lipopeptides. This hypothesis is derived from the multivariate linear regression analysis, where the OMs*LPs interaction showed an adverse effect on sporulation efficiency.

### Phr Mature Peptides, Critical Instruments for Modulation of Cannibalism and Sporulation in *B. velezensis* 83

Phr mature peptides, including the competence and sporulation stimulating factor “CSF” (PhrC mature peptide), are pentapeptides derived from the proteolytic processing of Phr pro-peptides. These mature peptides re-enter the cell through the ABC transporter system OppABCDF. Inside the cell, they act by inhibiting their respective cognate Rap response regulator aspartyl phosphatases, facilitating the process of sporulation [15, 24]. This occurs because the Rap proteins delay sporulation by inhibiting the phosphorylation of important regulators such as ComA, DegU, and Spo0A, in vegetative cells [24, 77]. The absence or presence of any of the Phr mature peptides affects sporulation. For example, *B. subtilis* mutant strains deficient in CSF production (∆*phrC*), exhibit a 50% reduction in sporulation compared to the parental strain, highlighting its importance [87, 95]. Consistently, exogenous CSF (PhrC mature peptide) added to a *B. subtilis* cell suspension induces *srfA* expression at low concentrations and triggers spore formation under nutrient-limited conditions at higher concentrations (with a low cell concentration) [87]. In contrast, the data presented here show that the exogenous addition of the synthetic CSF, equivalent to mature PhrC according to Stephenson et al., [90], results in a significant decrease in sporulation frequency compared to the control. Nonetheless, overall spore production was significantly higher, as shown by a notable increase in sporulation efficiency. Furthermore, the reduced half-sporulation time in this instance suggests a direct effect on cell synchronization, as expected. Interestingly, even with the addition of QS (synthetic CSF or lipopeptides) in nutrient-limiting in vitro scenarios, sporulation efficiency never surpassed 50%, and sporulation frequency never reached 100%. RapA phosphatase levels are heterogeneous across cells in a *Bacillus* population during the stationary phase. This heterogeneity modulates sporulation bistability [95],however, RapA alone only partially accounts for the phenomenon. Earlier studies have suggested that sporulation bistability is due to the collective action of several proteins involved in phosphorylation, such as the phosphatase Spo0E [95]. Transcriptomic studies in *B. velezensis* 83 have previously shown that the genes encoding the aspartate phosphatases RapA, RapF, RapC, and RapK, along with their corresponding inhibitors, the Phr-type peptides PhrF, PhrC, and PhrK (with the exception of PhrA, which is not present in the genome), are strongly up-regulated during the exponential phase [54]. This indicates that even with the induction of synthetic CSF, several Rap proteins may continue to inhibit sporulation. With the aim of deciding whether sporulation efficiency can still be improved, it would be interesting to conduct new experiments with mixtures of mature Phr peptides. Future studies also could explore the responses in the sporulation profiles of *B. velezensis* triggered by the evaluated factors (OMs, CSF, and LPs) and their connection to the transcription of cellular heterogeneity markers such as Rap-type phosphatases, with the purpose of identifying potential correlations that may facilitate the optimization of spore production processes, emphasizing critical process variables such as sporulation efficiency, and the proportion of spores with high germination capability.

In *Bacillus subtilis*, the *skfABCEFGH* and *sdpABC* operons have the coding sequences for the spore-killing factor SkfA and the toxin SdpC, which are responsible for cell lysis [26, 44, 49, 72]. This phenomenon leads to the loss of two-thirds of the cells, serving as a mechanism to recycle nutrients and delay sporulation when nutrients are limited. The transcription of these operons is triggered by Spo0A-P through the inhibition of AbrB, and only a part of the cells adopts the cannibalistic phenotype within the population [26, 49]. Lysis takes place in cells with reduced Spo0A-P levels, which do not produce the lysis factors SkfA and SdpC, nor their associated immunity systems [26, 44, 72]. The mode of action of SkfA remains unclear, while the toxin SdpC disrupts the proton motive force and triggers autolysis [44, 72]. Based on bioinformatic analysis, *B. velezensis* 83 contains only the orthologs of *sdpABC*. Considering that cells producing these toxins are immune to them, as occurs with the *sdpRI* operon responsible for SdpC immunity [26], it is plausible to hypothesize that the treatment involving the individual addition of CSF significantly increased the internal concentration of Spo0A-P, synchronizing the population and resulting in a significant decrease in the fraction of lysed cells.

Besides Spo0A-P-dependent cannibalistic factors, it is possible that additional mechanisms are involved in the observed cell autolysis, including normal cellular senescence and death, loss of the proton motive force, exposure to antibiotics, and pH changes [34, 80]. This hypothesis is plausible for cultures with resuspended cells in medium lacking carbon sources (e.g., negative control, 2^3^ design), where cannibalistic factors (e.g., SdpC) probably do not accumulate to levels high enough to induce the lysis of sensitive cells. Additionally, cell autolysis phenomenon might involve a more intricate dynamic. The initial cells that undergo autolysis as a result of normal cellular senescence and death (or other factors) likely provide resources for the remaining vegetative cells, progressively contributing to the accumulation of Spo0A-P-dependent cannibalistic factors and facilitating further cell autolysis in the non-immune cells.

### Sporulation Efficiency, as a Dependable Metric for Ensuring the Proper Subpopulation Balance During the Development of Processes in *B. velezensis* 83

Pioneering studies on quorum-sensing in *B. subtilis* reported that cultures at low cell concentrations (< 1 × 10^8^ cells/mL) exhibit a 90% or greater reduction in sporulation frequency compared to cultures with higher cell concentrations [28, 45, 87]. Comparatively, in the Glc_0_-/X_0_- condition, which had a maximum cell concentration of just 1 × 10^7^ cell/mL, the sporulation frequency decreased by only 22% relative to the average of the other three conditions (> 1 × 10^9^ cell/mL), without affecting sporulation efficiency. The observed discrepancy is likely a result of the short analysis period used in previous studies. In this work, the four conditions of 2^2^ experimental design and other experiments were evaluated until the sporulation frequency (the parameter historically most used) reached 90% or higher or until the spore concentration became constant. As a comparative example, Grossman and Losick [28] assessed only between 12 and 16 h after glucose depletion, a period too short to capture the completion of the process. In the Glc_0_-/X_0_- culture, ~ 2% sporulation frequency was detected after 18 h of nutrient limitation, with this value increasing to 67% over time. If the analysis had been conducted only between 12 and 16 h, the conclusion would have been identical. At the onset of nutrient limitation, higher cell concentrations favor spore formation, since the cell autolysis of a sufficient number of cells provides a greater resource pool, significantly influencing the distribution of subpopulations. For the optimization of subpopulation balance in *Bacillus* spore production, we suggest using sporulation efficiency as a key target variable. This parameter captures the relationship between the spore concentration and the maximum cell concentration reached in a culture, which considers the cell loss caused by lysis as part of the metric. Therefore, new strategies to reduce and/or prevent cannibalism should be addressed. Using this parameter, it was found that the Glc_0_-/X_0_- culture did not show deficient sporulation, as to those achieving cell densities two orders of magnitude higher. The primary distinction is that, when spore concentration levels off, approximately 20% of the population stays as vegetative cells, potentially due to insufficient signaling or inadequate energy supply for sporulation (Fig. 2). In another report, the addition of CSF in a range of 0.05 to 0.1 µM stimulated sporulation frequency by five- to tenfold in cultures with low cellular density (OD_600_ = 0.03, corresponding to approximately × 10^7^ cells/mL, since the exact value was not reported) after 12–14 h after nutrient limitation [45, 87]. While the role of CSF in promoting sporulation was emphasized, the very short observation period likely restricted the experiment to conditions with an extremely low sporulation frequency (control with FreqSpo = 0.1%), reinforcing the notion that low cell density inhibits sporulation [87], although this is not entirely accurate. In the current case with *B. velezensis* 83, the addition of CSF at 10 µM to a suspension containing × 10^9^ cells/mL, a specific concentration comparable to that employed by Solomon et al. [87], resulted in improved efficiency and a decreased proportion of lysed cells, but without significant changes in the final sporulation frequency (control with FreqSpo = 91%), according to the analysis performed up to the completion of the differentiation process (≈35 h post-nutrient depletion).

## Conclusion

Carbon overflow and quorum-sensing metabolites secreted during the growth phase of *B. velezensis* 83 significantly affect the sporulation profiles. The lipopeptides, surfactin, and bacillomycin D from *B. velezensis* 83 negatively influence sporulation efficiency, half-sporulation time, and sporulation interval. In contrast, the carbon overflow metabolites, acetoin and 2,3-BD, have completely opposite effects, increasing the levels of these three variables. CSF alone leads to a mixed effect, increasing sporulation efficiency but decreasing the duration of sporulation. Nevertheless, the interactions displayed by the evaluated factors led to complex and intricate responses, due to the various roles they play. From an ecological perspective, the generation and use of overflow metabolites would be a strategy to help both spore assembly and the maintenance of residual vegetative cells that did not surpass the Spo0A-P threshold during nutritional limitation scenarios, promoting a heterogeneous population with greater adaptability. Besides their recognized roles as surfactants that aid in colony expansion and as essential antimicrobial compounds involved in antagonism, according to the results presented here, lipopeptides would also serve as mediators of cellular differentiation pathways by enhancing synchronization. Sporulation in *B. velezensis* 83 is a highly regulated and complex event, in which metabolites generated during the growth phase (OM, CSF, and LPs) perform complementary roles in terms of efficiency and synchronization.

## Supplementary Information

Below is the link to the electronic supplementary material.

Supplementary file1 (DOCX 42 KB)

## Data Availability

No datasets were generated or analysed during the current study.
