# Supplementary material for: Modulation of the Sporulation Dynamics in the Plant-Probiotic Bacillus velezensis 83 via Carbon and Quorum-Sensing Metabolites
Source: Probiotics Antimicrob Proteins. 2025 Feb 26;17(5):3743–62. doi: 10.1007/s12602-025-10482-w (PMC12532671; doi:10.1007/s12602-025-10482-w)
Supplement: Supplementary file 1 — Supplementary file1 (DOCX 42 KB) [file 12602_2025_10482_MOESM1_ESM.docx]

**Supplementary Information for:**

**Modulation of the sporulation** **dynamics in the plant-probiotic *Bacillus velezensis* 83 via carbon and *quorum-sensing* metabolites**

**Esmeralda Yazmín Soriano-Peña**^†^, **Agustín Luna-Bulbarela**^†^, **Sergio Andrés Cristiano-Fajardo**, **Enrique Galindo***, **Leobardo Serrano-Carreón***

Departamento de Ingeniería Celular y Biocatálisis, Instituto de Biotecnología, Universidad Nacional Autónoma de México, Av. Universidad 2001, C.P.62210, Cuernavaca, Morelos, México.

^†^These two authors contributed equally to this work.

*Corresponding authors: 52 (777) 329-1630, *E-mail*: leobardo.serrano@ibt.unam.mx; enrique.galindo@ibt.unam.mx

**Table of Contents:**

| **Table 1S** | 2^3^ Factorial experimental design to assess the influence of carbon overflow metabolites (OMs), Competence/Sporulation Stimulating Factor (CSF), and lipopeptides (LPs) on sporulation efficiency (%Eff_Spo_), half-sporulation time (t_1/2 Spo_), and sporulation interval (I_Spo_) in *B. velezensis* 83. | Page 2 |
| --- | --- | --- |
| **Table 2S** | Effect of OMs, CSFs, and LPs on half-sporulation time. | Page 3 |
| **Table 3S** | Effect of OMs, CSFs, and LPs on sporulation interval. | Page 4 |

**Table 1S. 2^3^ Factorial experimental design to assess the influence of carbon overflow metabolites (OMs), Competence and Sporulation Stimulating Factor (CSF), and lipopeptides (LPs) on sporulation efficiency (%Eff_Spo_), half-sporulation time (t_1/2 Spo_), and sporulation interval (I_Spo_) in *B. velezensis* 83.** Values for statistical analysis were normalized to Glc_0_+/X_0_- condition. The model uses the following coded levels for each of the factors: -1, 0, and +1. These levels denote absence, half the concentration, and the concentration observed in Glc_0_+/X_0_- condition 1-hour post-glucose depletion, respectively. Data includes model-calculated and experimental data from shaken flask trials. Coefficient of correlation (R^2^) of the model is provided in parentheses.

| **Factor** | | |  | **Responses** | | | | | | | |
| --- | --- | --- | --- | --- | --- | --- | --- | --- | --- | --- | --- |
| **OMs** | **CSF** | **LPs** |  | **%Eff_Spo_(0.93)** | |  | **t_1/2 Spo_ (0.84)** | |  | **I_Spo_ (0.78)** | |
|  |  |  |  | **Model** | **Experimental** |  | **Model** | **Experimental** |  | **Model** | **Experimental** |
|  |  |  |  |  | 0.61 |  |  | 1.89 |  |  | 1.3 |
| **-1** | **-1** | **-1** |  | **0.64** | 0.64 |  | **1.53** | 1.89 |  | **1.20** | 1.3 |
|  |  |  |  |  | 0.61 |  |  | 1.86 |  |  | 1.3 |
|  |  |  |  |  | 1.29 |  |  | 3.21 |  |  | 8.0 |
| **1** | **-1** | **-1** |  | **1.22** | 1.18 |  | **2.91** | 3.10 |  | **6.80** | 8.0 |
|  |  |  |  |  | 1.14 |  |  | 2.95 |  |  | 7.3 |
|  |  |  |  |  | 1.05 |  |  | 1.78 |  |  | 2.0 |
| **-1** | **1** | **-1** |  | **1.04** | 1.03 |  | **1.53** | 1.62 |  | **1.20** | 2.0 |
|  |  |  |  |  | 0.99 |  |  | 1.65 |  |  | 2.0 |
|  |  |  |  |  | 0.92 |  |  | 3.01 |  |  | 7.3 |
| **1** | **1** | **-1** |  | **1.01** | 0.97 |  | **2.91** | 3.03 |  | **6.80** | 7.3 |
|  |  |  |  |  | 1.09 |  |  | 3.16 |  |  | 7.3 |
|  |  |  |  |  | 0.49 |  |  | 1.63 |  |  | 2.3 |
| **-1** | **-1** | **1** |  | **0.52** | 0.50 |  | **1.53** | 1.54 |  | **1.20** | 1.7 |
|  |  |  |  |  | 0.53 |  |  | 1.59 |  |  | 2.3 |
|  |  |  |  |  | 0.49 |  |  | 2.84 |  |  | 7.3 |
| **1** | **-1** | **1** |  | **0.52** | 0.54 |  | **2.91** | 2.91 |  | **6.80** | 7.3 |
|  |  |  |  |  | 0.49 |  |  | 2.98 |  |  | 7.3 |
|  |  |  |  |  | 0.8 |  |  | 1.42 |  |  | 1.3 |
| **-1** | **1** | **1** |  | **0.76** | 0.66 |  | **1.53** | 1.36 |  | **1.20** | 1.3 |
|  |  |  |  |  | 0.77 |  |  | 1.43 |  |  | 1.3 |
|  |  |  |  |  | 0.63 |  |  | 3.03 |  |  | 7.3 |
| **1** | **1** | **1** |  | **0.60** | 0.57 |  | **2.91** | 2.98 |  | **6.80** | 6.7 |
|  |  |  |  |  | 0.55 |  |  | 2.96 |  |  | 6.7 |
|  |  |  |  |  | 0.81 |  |  | 1.69 |  |  | 2.0 |
|  |  |  |  |  | 0.87 |  |  | 1.72 |  |  | 1.3 |
| **0** | **0** | **0** |  | **0.79** | 0.97 |  | **2.22** | 1.72 |  | **4.00** | 1.3 |
|  |  |  |  |  | 0.89 |  |  | 1.75 |  |  | 1.3 |
|  |  |  |  |  | 0.83 |  |  | 1.70 |  |  | 1.3 |

**Table 2S. Effect of OMs, CSF, and LPs on half-sporulation time**. The table shows the analysis of variance and multivariable regression of the 2^3^-factorial design. The impact of carbon overflow metabolites (OMs), Competence/Sporulation Stimulating Factor (CSF), and lipopeptides (LPs) or their interactions on half-sporulation time in *B. velezensis* 83 is present through a linear model. The corresponding data were obtained by shaken flask trials. Values for statistical analysis were normalized to Glc_0_+/X_0_- condition. The significant factors and interactions are indicated in blue. The model uses the following coded levels for each of the factors: -1, 0, and +1. These levels denote absence, half the concentration, and the concentration observed in Glc_0_+/X_0_- condition 1-hour post-glucose depletion, respectively.

| **Source** | **Sum of**  **squares** | **DF** | **Mean**  **square** | **F value** | **p-value**  **Prob > F** | **Coefficient**  **estimate** |
| --- | --- | --- | --- | --- | --- | --- |
| Model | 11.76 | 7 | 1.68 | 21.74 | < 0.0001 | **β_0_ = 2.22** |
| **OMs** | 11.34 | 1 | 11.34 | 146.8 | < 0.0001 | **β_1_ = 0.69** |
| **CSF** | 0.038 | 1 | 0.038 | 0.50 | 0.4886 | **β_2_ = -0.040** |
| **LPs** | 0.26 | 1 | 0.26 | 3.32 | 0.0829 | **β_3_ = -0.10** |
| **OMs*CSF** | 0.073 | 1 | 0.073 | 0.94 | 0.3434 | **β_4_ = 0.055** |
| **OMs*LPs** | 0.038 | 1 | 0.038 | 0.50 | 0.4886 | **β_5_ = 0.040** |
| **CSF*LPs** | 0.004817 | 1 | 0.004817 | 0.062 | 0.8053 | **β_6_ = 0.014** |
| **OMs*CSF*LPs** | 0.002817 | 1 | 0.002817 | 0.036 | 0.8504 | **β_7_ = 0.011** |
| Residual | 1.62 | 21 | 0.077 |  |  |  |
| Lack of Fit | 1.54 | 1 | 1.54 | 367.04 | < 0.0001 |  |
| Pure Error | 0.084 | 20 | 0.004193 |  |  |  |
| Cor Total | 13.38 | 28 |  |  |  |  |
| R-Squared | 0.84 |  |  |  |  |  |
| **Linear Model:**  **t_1/2 Spo_ = + β_0_ + β_1_OMs**  **t_1/2 Spo_ = + 2.22 + 0.69OMs** | | | | | | |

**Table 3S. Effect of OMs, CSF, and LPs on sporulation interval**. The table shows the analysis of variance and multivariable regression of the 2^3^-factorial design. The impact of carbon overflow metabolites (OMs), Competence/Sporulation Stimulating Factor (CSF), and lipopeptides (LPs) or their interactions on sporulation interval in *B. velezensis* 83 is present through a linear model. The corresponding data were obtained by shaken flask trials. Values for statistical analysis were normalized to Glc_0_+/X_0_- condition. The model uses the following coded levels for each of the factors: -1, 0, and +1. These levels denote absence, half the concentration, and the concentration observed in Glc_0_+/X_0_- condition 1-hour post-glucose depletion, respectively.

| **Source** | **Sum of**  **squares** | **DF** | **Mean**  **square** | **F value** | **p-value**  **Prob > F** | **Coefficient**  **estimate** |
| --- | --- | --- | --- | --- | --- | --- |
| Model | 193.80 | 7 | 27.69 | 14.59 | < 0.0001 | **β_0_ = 3.97** |
| **OMs** | 190.97 | 1 | 190.97 | 100.66 | < 0.0001 | **β_1_ = 2.82** |
| **CSF** | 0.35 | 1 | 0.35 | 0.18 | 0.6717 | **β_2_ = -0.12** |
| **LPs** | 0.22 | 1 | 0.22 | 0.12 | 0.7366 | **β_3_ = -0.096** |
| **OMs*CSF** | 0.22 | 1 | 0.22 | 0.12 | 0.7366 | **β_4_ = -0.096** |
| **OMs*LPs** | 0.35 | 1 | 0.35 | 0.18 | 0.6717 | **β_5_ = -0.12** |
| **CSF*LPs** | 0.77 | 1 | 0.77 | 0.41 | 0.5308 | **β_6_ = -0.18** |
| **OMs*CSF*LPs** | 0.92 | 1 | 0.92 | 0.49 | 0.4937 | **β_7_ = 0.20** |
| Residual | 39.84 | 21 | 1.90 |  |  |  |
| Lack of Fit | 38.64 | 1 | 38.64 | 644.72 | < 0.0001 |  |
| Pure Error | 1.20 | 20 | 0.060 |  |  |  |
| Cor Total | 233.64 | 28 |  |  |  |  |
| R-Squared | 0.781 |  |  |  |  |  |
| **Linear Model:**  **I_Spo_ = + β_0_ + β_1_OMs**  **I_Spo_ = + 3.97 + 2.82OMs** | | | | | | |
